# Predicting Type 2 diabetes onset age using machine learning: A case study in KSA

**DOI:** 10.1371/journal.pone.0318484

**Published:** 2025-02-11

**Authors:** Faten Al-hussein, Laleh Tafakori, Mali Abdollahian, Khalid Al-Shali, Ahmed Al-Hejin

**Affiliations:** 1 School of Science, RMIT University, Melbourne, Victoria, Australia; 2 Department of Mathematics and Statistics, College of Sciences, University of Jeddah, Jeddah, Saudi Arabia; 3 Department of Medicine, King Abdulaziz University Hospital, Jeddah, Saudi Arabia; 4 Department of Biological Sciences, Faculty of Science, King Abdulaziz University, Jeddah, Saudi Arabia; University 20 Aout 1955 skikda, Algeria, ALGERIA

## Abstract

The rising prevalence of Type 2 Diabetes (T2D) in Saudi Arabia presents significant healthcare challenges. Estimating the age at onset of T2D can aid early interventions, potentially reducing complications due to late diagnoses. This study, conducted at King Abdulaziz Medical University Hospital, aims to predict the age at onset of T2D using Multiple Linear Regression (MLR), Artificial Neural Networks (ANN), Random Forest (RF), Support Vector Regression (SVR), and Decision Tree Regression (DTR). It also seeks to identify key predictors influencing the age at onset of T2D in Saudi Arabia, which ranks 7th globally in prevalence. Medical records from 1,000 diabetic patients from 2018 to 2022 that contain demographic, lifestyle, and lipid profile data are used to develop the models. The average onset age was 65 years, with the most common onset range between 40 and 90 years. The MLR and RF models provided the best fit, achieving R^2^ values of 0.90 and 0.89, root mean square errors (RMSE) of 0.07 and 0.01, and mean absolute errors (MAE) of 0.05 and 0.13, respectively, using the logarithmic transformation of the onset age. Key factors influencing the age at onset included triglycerides (TG), total cholesterol (TC), high-density lipoprotein (HDL), ferritin, body mass index (BMI), systolic blood pressure (SBP), white blood cell count (WBC), diet, and vitamin D levels. This study is the first in Saudi Arabia to employ MLR, ANN, RF, SVR, and DTR models to predict T2D onset age, providing valuable tools for healthcare practitioners to monitor and design intervention strategies aimed at reducing the impact of T2D in the region.

## 1. Introduction

Type 2 Diabetes (T2D) has emerged as a significant global health crisis, with its prevalence rising rapidly and being recognized as one of the leading causes of adult mortality in the 21st century [1]. This condition occurs when blood sugar (glucose) levels become abnormally elevated, leading to serious health complications such as heart and kidney diseases if not properly managed [2,3]. The World Health Organization (WHO) defines diabetes as a metabolic disorder of various causes, characterized by chronic high blood sugar levels with disruption of carbohydrate, fat, and protein metabolism due to defects in insulin secretion, insulin action, or both [4].

In 2023 the global diabetic population exceeded 500 million, and it is projected to surpass 1.3 billion over the next three decades, representing a growth rate of more than double [5]. According to the International Diabetes Federation (IDF), data from 2021 indicates that approximately 537 million adults (aged 20–79 years) are living with diabetes worldwide, representing 1 in 10 adults. This number is expected to rise to 643 million by 2030, with 1 in 9 adults affected, and further increase to 784 million by 2045, with 1 in 8 adults impacted [6]. Diabetes is also projected to become the seventh leading cause of mortality worldwide by 2030 [7].

Saudi Arabia is ranked among the countries with the highest T2D prevalence rates globally and the fourth highest in the Middle East and North Africa region [8]. Saudi Arabia experienced a significant increase in the number of diabetes cases compared to 2015, with an additional 10 million people diagnosed, 34 million more at risk of developing diabetes, and 19 million undiagnosed cases. This comparison is based on the most recent data available at the time of the study, which corresponds to data from 2017 [7]. This data raises significant concerns about the growing health and economic burdens associated with this disease in Saudi Arabia [7].

The IDF warns that Saudi Arabia could face one of the most severe global scenarios, where half of its population may suffer from diabetes by 2030 if effective preventive measures are not implemented [9]. Furthermore, several sources describe the widespread prevalence of diabetes in Saudi Arabia as an “epidemic,” emphasizing the urgent need for intervention to mitigate its impact [7,10,11].

Environmental and lifestyle risk factors, which vary significantly based on geographical location, account for the differences in incidence rates worldwide, with the Middle East experiencing a projected 87% increase by 2045, reaching 136 million people, while Europe sees a 24% rise, reaching 69 million people by the same year [6,12]. Despite the increasing prevalence of T2D in Saudi Arabia, research on this condition remains significantly underrepresented compared to studies conducted in more developed nations [11]. To date, no comprehensive study has been conducted across Saudi Arabia addressing the age at onset and related risk factors for T2D. Moreover, previous studies conducted in the country often share a common limitation of being cross-sectional design with small sample sizes and limited variables, which are insufficient to represent the diverse demographics of Saudi Arabia [13,14]. This research gap underscores the need for targeted studies on the age at onset of T2D, as this could lead to improved diagnostic timing and intervention efficacy both nationally and globally.

Despite these alarming statistics, diabetes remains the most widespread chronic disease in Saudi Arabia, with insufficient emphasis placed on predicting the age at the onset of T2D. Enhancing our understanding of the development of T2D among Saudis is crucial to improve monitoring and reduce complications caused by late diagnosis. Identifying the age at which this condition initiates may enhance the practicality of early intervention strategies designed to postpone the disease progression, thereby safeguarding a greater number of active pancreatic cells before the onset of significant glycation damage. This can lead to lifestyle and therapeutic changes that may delay or prevent the progression of the disease.

The present investigation seeks to fill the shortage in the literature by providing the best predictive model of the age at the onset of T2D in the Saudi population and identifying its most significant risk factors. This study explores the performances of local data-based Multiple Linear Regression (MLR), Artificial Neural Networks (ANN), Random Forest (RF), Support Vector Regression (SVR), and Decision Tree Regression (DTR).

methods for estimating the age at onset of T2D for individuals aged 20 years and above. To the best of our knowledge, none of these machine learning models have ever been applied in the context of Saudi Arabia to predict the age at onset of T2D; thus, our research makes an important contribution to the current body of literature on predicting diabetes in the region.

## 2. Literature review

T2D is a complicated disease impacted by various variables, including modifiable and non-modifiable risk factors, such as age, obesity, sex, lipid profiles, and lifestyle habits [15]. Understanding these risk factors is essential in delaying or even preventing the onset of T2D. Among these features, age is especially significant since it is crucial in the prognosis and therapy of the disease. Several international guidelines reflect this importance; for instance, the American Diabetes Association recommends T2D screening for individuals aged 35 and above without risk factors, while guidelines in Singapore suggest screening at age 40 [16,17].

The age at onset of T2D can significantly influence the likelihood of subsequent complications. Multiple studies have examined this association, reporting different outcomes based on population characteristics. Some studies [18–20] suggest that earlier onset of T2D can lead to a higher risk of complications, as the disease remains active for a long period. Prolonged exposure to elevated blood sugar levels can damage the kidneys or blood vessels. On the other hand, other studies [21,22] argue that earlier diagnosis may reduce the risk of complications by providing an opportunity for early intervention, such as lifestyle modifications and better glycemic control, which can slow down the progression of the disease. Meanwhile, another study [23] has found no clear relationship or statistically significant difference between the age of diagnosis and the risk of complications, indicating that the outcomes may depend on other factors, such as the quality of healthcare or the patient’s adherence to treatment. Furthermore, age at diagnosis may affect specific body systems differently [24,25], demonstrating the complex interaction between age and diabetes-related health outcomes.

Ethnicity significantly influences the age-related prevalence of T2D, adding a layer of complexity to data interpretation. A comparative study revealed that European populations tend to experience elevated diabetes rates later in life compared to East Asian groups, such as Chinese and Japanese. These differences persisted even after controlling for body mass index (BMI), which is considered one of the key factors in early disease detection [26].

A study conducted by the U.S. Health Maintenance Organization, using data on adults newly diagnosed with T2D between 1996 and 1998, revealed that younger individuals were more likely to develop the disease, primarily due to higher rates of obesity among them. Their average body mass index (BMI) was 39 kg/m^2^ compared to 33 kg/m^2^ for older individuals (P < 0.001). The study also showed that the majority of younger patients were female (P = 0.04) [27].

Region-specific studies further emphasize the role of localized risk factors in T2D onset. A study conducted at King Saud University Diabetes Center in Riyadh, Saudi Arabia, identified several key risk factors, including fasting glucose level (95% CI: 1.051–1.273, Risk Ratio: 1.157), triglyceride level (95% CI: 1.086–1.538, Risk Ratio: 1.290), LDL level (95% CI: 1.073–1.574, Risk Ratio: 1.299), cholesterol level (95% CI: 1.174–2.147, Risk Ratio: 1.588), and male gender (95% CI: 1.090–1.536, Risk Ratio: 1.294) [28]. Another study conducted in northeastern Iran also identified age and white blood cell (WBC) count as significant predictors of T2D [29]. In a study conducted in China involving individuals aged 45 and above, risk factors for T2D were found to include low levels of high-density lipoprotein cholesterol (HDL-C), elevated cholesterol (hypercholesterolemia), and triglycerides (hypertriglyceridemia) [30].

Researchers have demonstrated the effectiveness of machine learning models in predicting the age at onset of T2D. Studies conducted in Taiwan and Pakistan to predict diabetes onset using machine learning methods showed that the methods performed well [31,32]. A study conducted in Slovenia applied machine learning techniques and linear regression. Their results showed that the random forest (RF) model outperformed others [33]. Researchers in Taiwan utilised different machine learning techniques to predict diabetes risk and facilitate early detection. They concluded that the random forest (RF) and artificial neural networks (ANN) algorithms produced effective results. In particular, the RF algorithm achieved a classification accuracy of 88.31% [34]. A study conducted in India using the PIMA Indian dataset tested several machine learning algorithms including logistic regression (LR), XGBoost (XGB), gradient boosting (GB), decision trees (DT), extra trees (ET), random forest (RF), and light gradient boosting machine (LGBM) to predict T2D onset. The LGBM classifier outperformed others with an accuracy of 95.20% [35].

In addition, previous studies have shown that machine learning algorithms have significant potential in predicting the age of onset in other diseases. In a study conducted in Saudi Arabia, multiple linear regression (MLR) and supervised machine learning techniques were employed to predict the age of onset for type 1 childhood diabetes. The MLR and RF demonstrated high predictive performance, with R^2^ values of 0.88 and 0.89, respectively, and low RMSE values of 0.22 and 0.21, alongside MAE values of 0.18 and 0.17 [36]. Another study conducted in Saudi Arabia focused on predicting the age of obesity onset among children and adolescents. Four predictive models were employed: multiple linear regression (MLR), random forest (RF), decision tree (DT), and k-nearest neighbors (KNN). The results revealed that RF outperformed the other models, achieving an R^2^ value of 0.9844, an RMSE of 0.44, and an MAE of 0.28, followed by MLR, DT, and KNN [37]. These findings underscore the pivotal role of machine learning models, particularly RF, in enhancing predictive accuracy for various health conditions.

Traditional statistical methods such as regression models have also been widely used to predict the likelihood of developing T2D. One notable study employed logistic regression (LR) to model the risk of T2D onset and showed significant correlations between age, fasting plasma glucose, and family history of diabetes [38].

The literature review summarised above indicates an imminent need for specialized studies on the association of age at the onset of T2D with various risk factors such as lifestyle and environment within the Saudi population. The present study attempts to fill a part of this gap by investigating the interaction of age at the onset of T2D with various risk factors in the Saudi community through advanced regression and machine learning models.

## 3. Materials and methodology

This section delineates the comprehensive methodologies employed for data collection, model development, and result validation. Data was meticulously gathered and analyzed to construct predictive models to estimate the age at onset of T2D. We employed statistical techniques and advanced machine learning, ensuring each model was rigorously tested for predictive accuracy and reliability.

The models utilized in this research include MLR, ANN, RF, SVR, and DTR. These models were meticulously selected based on their proven effectiveness in addressing similar analytical challenges within medical contexts. The previous studies [31–37] have demonstrated their ability to handle complex data for predicting the onset age of diabetes and other diseases, ensuring robust analysis in identifying the key factors influencing the age at onset of T2D.

### 3.1. Data collection

Data were obtained from medical records of 4,526 patients aged over 20 years from King Abdulaziz University Hospital (KAUH), Jeddah, Saudi Arabia. The research received approval from the Human Research Ethics Committee at RMIT University in Australia and the Research Ethics Committee at KAUH. This study was retrospective as the analysis was of the observed pre-existing medical records from KAUH; all data was anonymized before analysis. The analysis did not need informed consent from the ethics review committee because the research would be done using previously obtained medical data. Access to the data was given in 19/11/2023 after ethical clearance was approved. The sample includes patients who were diagnosed with T2D between 01/01/2018 and 31/12/2022.

The dataset consisted of both categorical and numerical variables. The categorical variables in the dataset included gender, nationality, smoking status, physical activity, type of food, and hypertension (all Yes/No responses). Categorical data included occupation, employed/unemployed, and marital status (married/unmarried). Numerical variables included age, body mass index (BMI), total cholesterol (TC), triglycerides (TG), high-density lipoprotein (HDL), systolic blood pressure (SBP), diastolic blood pressure (DBP), vitamin D, white blood cell count (WBC), glycated hemoglobin (HbA1c) and ferritin level. Descriptive statistics for the numerical variables and the p-value corresponding to categorical variables have been provided in Table 1.

**Table 1:** Descriptive statistics for the numerical variables and the p-value corresponding to categorical variables (n = 1000).

ID	Feature Name	Count (N = 1000), n (%)	t	p-value
**Categorical Variables**				
1	Gender	Female:446(44.56%) Male: 554(55.44%)	−0.89	0.369
2	BMI	Underweight:22(2.2%); Normal weight:184(18.47%); Overweight:337(33.77%); Obese:457(45.7%)	Underweight vs Normal weight = −26.26 Underweight vs Overweight = −52.63 Underweight vs Obese = −86.02 Normal weight vs Overweight = −52.25 Normal weight vs Obese = −92.90 Overweight vs Obese = −67.40	0.000^*^
3	Marital Status	Married:818 (82%) Not Married:182(18.2%)	6.62	0.056
4	Nationality	Saudia Arabia:639 (63.9%) Not Saudia Arabia: 361 (36.1%)	3.13	0.033^*^
5	Occupation	Employed: 288 (28.8%) Not Employed: 712 (71%)	−5.19	0.000^*^
6	Smoking	Yes:869 (86.9%) No:131 (13.1%)	−1.07	0.285
7	Physical Activity	Yes:871 (87.1%) No:129 (12.9%)	−0.57	0.057
8	Type of Food	Heathy:351 (35.1%) Non healthy:649 (64.9%)	−3.78	0.000^*^
9	Hypertension	Yes:782 (78%) No:218 (21.8%)	−3.71	0.000^*^
10	WBC	Low: 46 (4.6%) Good: 458 (45.8%) High: 496 (49.6%)	Low vs Good = −3.25 Low vs High = −3.62 Good vs High = −7.29	0.000^*^ 0.023^*^ 0.000^*^
11	HDL	Low: 585 (58.5%) Good: 224 (22.4%) High: 191 (19.1%)	Low vs Good = −6.58 Low vs High = −4.17 Good vs High = −3.97	0.000^*^ 0.002^*^ 0.035^*^
12	TC	Normal: 223 (22.3%) Moderately high: 206 (20.6%) High: 571 (57.1%)	Normal vs Moderately high = −3.79 Normal vs High = −2.90 High vs Moderately high = −3.51	0.000^*^ 0.004^*^ 0.013^*^
13	TG	Normal: 198 (19.8%) Moderately high: 206 (20.6%) High: 596 (59.6%)	Normal vs Moderately high = −9.77 Normal vs High = −21.88 High vs Moderately high = −7.57	0.000^*^ 0.000^*^ 0.000^*^
14	Vitamin- D	Deficient: 478 (47.7%) Insufficient: 231 (23.1%) Sufficient: 291 (29.1%)	Deficient vs Insufficient = −7.19 Deficient vs Sufficient = −3.59 Sufficient vs Insufficient = −11.42	0.023^*^ 0.022^*^ 0.000^*^
15	Ferritin	Deficient:581(58.1%) Insufficient::230(23%) Sufficient:189(18.9%)	Deficient vs Insufficient = 4.10 Deficient vs Sufficient = −7.09 Sufficient vs Insufficient = −3.32	0.011^*^ 0.022^*^ 0.013^*^
16	Systolic blood pressure	Low:166(16.58%) Normal:381(38.1%) High:453(45.25%)	Low vs Normal = −3.14 Low vs High = −8.79 Normal vs High = −14.80	0.002^*^ 0.000^*^ 0.000^*^
17	Diastolic blood pressure	Low:668(66.8%) Normal:181(18.08%) High:151(15.09%)	Low vs Normal = −5.11 Low vs High = −7.35 Normal vs High = −5.34	0.000^*^ 0.000^*^ 0.000^*^
**Numerical Variables**				
18	Age	Max: 104, Min:20, Median: 65, Mean: 65, SD: 10		
19	Weight	Max: 175, Min: 35, Median: 77, Mean: 75, SD: 19		
20	Height	Max: 188, Min: 109, Median: 163, Mean: 163.11, SD: 13.17		
21	HbA1c	Max: 18.9, Min: 6.5, Median: 9.00, Mean: 9.54, SD: 2.37		

(*) Indicates statistically significant differences.

For classification by BMI, WHO guidelines [39,40] were followed: underweight (<18.5 kg/m^2^), normal weight (18.5–24.9 kg/m^2^), overweight (25.0–29.9 kg/m^2^), and obese (≥30.0 kg/m^2^). HbA1c levels were classified as normal (HbA1c < 6.5%) and newly diagnosed diabetes (HbA1c ≥ 6.5%) according to IDF guidelines [41]. The lipid profile was then further categorized into HDL as being either low (<40 mg/dL for males and < 50 mg/dL for females), good (40–59 mg/dL for males and 50–59 mg/dL for females), and high (≥60 mg/dL for both sexes) [42]. TC was classified into desirable (<200 mg/dL), moderately high (200–239 mg/dL), and high (≥240 mg/dL); levels of TGs were classified into normal (<149 mg/dL), moderately high (150–199 mg/dL), and high (≥200 mg/dL) [42]. The levels of vitamin D were divided into three categories: deficient (<30 ng/mL), insufficient- (30–50 ng/mL), and sufficient- (≥50 ng/mL) [43]. Ferritin levels were compared to the normal range; the normal values were between 24–336 micrograms per litter for men and 11–307 micrograms per litter for women. Values outside this range were further classified as either low, which could represent iron deficiency or anemia, or high [44], as detailed in Table 1.

Data were filtered in two steps to ensure data quality. First, variables were excluded for which data on more than 90% of the patients in the dataset were incomplete. This was followed by removing the variables that showed negligible variation across more than 90% of the cohort. This process reduced the number of variables from 35 to 21 and the number of records from 4,526 to 1,000 patients.

**Fig 1:**
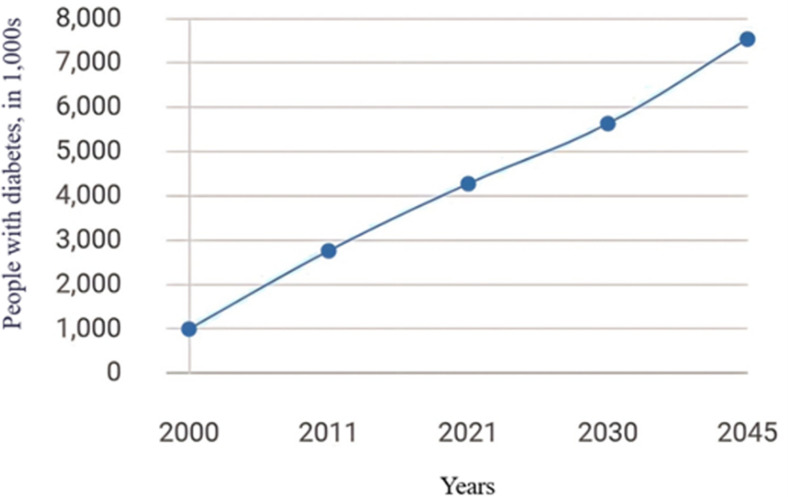
Trends in Estimated T2D in Saudi Arabia (2000–2045).

**Fig 2:**
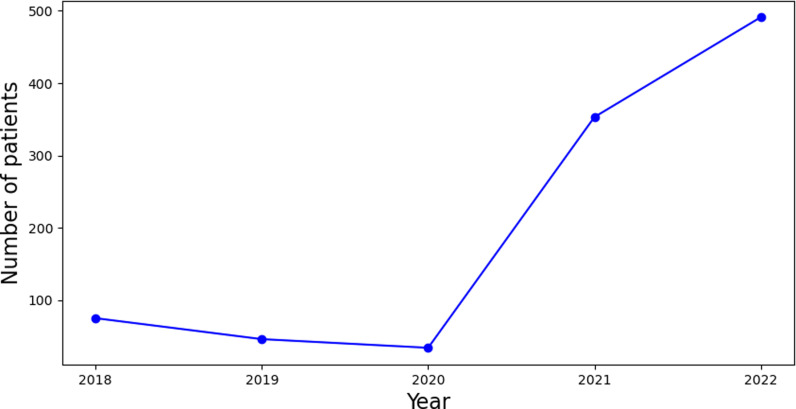
Number of patients in KAUH between (2018–2022).

The IDF [45] projects that the number of individuals with diabetes in Saudi Arabia would increase from over 1 million in 2000 to over 7 million by 2045, as seen in Fig 1. The IDF projects that in the next years the number of cases will keep increasing. A comprehensive review of the records during the last five years has demonstrated a notable rise in T2D diagnoses at KAUH in Jeddah, Saudi Arabia, as illustrated in Fig 2. The data indicates that the frequency of reported cases has risen in these facilities during the 2018–2022 period.

### 3.2. Model development

This study examines the complete cohort and the age range of 40–90 years, identified as the most prevalent interval for the age at onset of T2D. The goal was to establish a model for the age at onset of T2D by utilizing a series of predictive factors recognized in previous research. Earlier studies [46–52] indicated that factors including gender, weight, height, body mass index (BMI), occupation, smoking, marital status, physical activity, hypertension, blood pressure, high-density lipoprotein (HDL), HbA1c levels, triglycerides (TG), total cholesterol (TC), white blood cell (WBC), and vitamin D at diagnosis can affect the age at onset of T2D. Consequently, these factors were integrated into the analysis for model development.

Various assessment criteria were utilised to evaluate the models’ performance, including the coefficient of determination (R^2^), root mean square error (RMSE), and mean absolute error (MAE). These metrics allowed for a comprehensive comparison of the model’s predictive accuracy. MLR, ANN, RF, SVR, and DTR were selected for this study, as they have been widely used in similar studies to model complex systems [53–58]. Each model was built and validated using Python as the statistical tool [59], allowing for efficient computation and analysis across the dataset.

#### 3.2.1. Multiple linear regression (MLR).

This study utilizes MLR [60] as a predictive technique to model age at onset of T2D. The model incorporates both independent factors identified from earlier studies and extra variables collected in this analysis. The MLR model is defined by the following equation:


$$y=\beta_{0}+\beta_{1}x_{1}+\beta_{2}x_{2}+\ldots +\beta_{k}x_{k}+\epsilon$$ (1)

where y represents the dependent variable (age at onset), $\beta_{0}$ is the intercept, $\beta_{1},\ldots ,\beta_{k}$ are the regression coefficients corresponding to the independent variables or interaction terms, $x_{1},\ldots ,x_{k}$ denote the independent variables (predictors), and ∊ is the error term of the model.

#### 3.2.2. Artificial Neural Network (ANN).

ANN are widely utilised in medical research for tasks such as data classification and predicting medical outcomes, significantly enhancing the accuracy of diagnoses and treatments [61]. These networks are capable of learning patterns from data without the need for predefined assumptions, making them well-suited for recognizing both linear and nonlinear relationships within complex datasets [62,63]. In this study, ANN comprises input layers that capture relevant risk factors for T2D onset, hidden layers that process these inputs through connection weights, and output layers that produce the predictive outcomes [64]. The connection weights between layers are iteratively updated based on the errors calculated during the training process, leading to improved prediction accuracy [65]. ANN’s flexibility in handling large datasets with intricate variable interactions makes it highly effective for modeling T2D onset.

#### 3.2.3. Random Forest (RF).

RF is a prevalent ensemble learning technique that constructs multiple decision trees from random samples drawn from the dataset, combining the results to enhance forecasting precision and dependability [66,67]. The model selects the most frequent prediction among individual trees, reducing the risk of overfitting and effectively handling a large number of input variables [68,69]. RF is particularly advantageous in medical research because it includes an inherent feature selection mechanism, identifying the most influential variables for predicting T2D onset [70,71]. In this study, RF’s ability to manage complex, high-dimensional data makes it a suitable model for analyzing the diverse factors influencing the age at onset of T2D.

#### 3.2.4. Support Vector Regression (SVR).

SVR is a machine learning technique employed to predict continuous outcomes, such as the age at onset of T2D. SVR establishes a decision boundary, or hyperplane, that optimises the margin among the closest points of data, known as support vectors, ensuring robust predictions [72]. The kernel approach in SVR enables the modeling of both linear and nonlinear relationships by transforming data into higher-dimensional spaces [73–75]. The choice of kernel, along with parameters such as cost and gamma, plays a critical role in controlling model performance and overfitting. These parameters are optimized using cross-validation to enhance prediction accuracy [73]. SVR’s ability to handle complex, nonlinear data makes it a powerful tool for predicting the onset of multifactorial conditions like T2D.

#### 3.2.5. Decision Tree Regression (DTR).

DTR is a straightforward yet powerful model for predicting continuous outcomes based on decision-making rules derived from the data [76]. The model divides the dataset into increasingly smaller groups based on significant predictors, with each node representing a decision point and the final prediction made at the terminal nodes [77,78]. In this study, DTR is utilized to predict the age at onset of T2D by evaluating variables such as BMI, lipid profiles, and blood pressure. The model’s accuracy is evaluated using the mean squared error (MSE), which measures the difference among the expected and actual results [79]. DTR is highly valued in medical research for its interpretability and ability to provide clear insights into how different variables influence outcomes.

## 4. Results analysis

Table 1 indicates that males (55.44%) are more likely to developing T2D than females (44.56%). In terms of BMI, 45.7% of the participants are categorized as obese, emphasizing obesity as a major risk factor. Smoking is prevalent, with 86.9% of the sample identified as smokers. Regarding physical activity, 87.1% of the participants engage in regular physical activity. However, the mean HbA1c level is recorded at 9.54%, indicating concerningly high blood sugar levels. In terms of lipid variables, the majority of participants exhibit elevated TC and TG levels, while many have low HDL levels. Vitamin D deficiency affects 47.8% of the participants, potentially exacerbating their health risks. WBC is another critical indicator, as deviations from normal counts may signal underlying health issues. Furthermore, abnormal ferritin levels, indicative of iron storage, are observed in a substantial portion of the sample. Lastly, elevated SBP and DBP readings further increase the risk of complications.

The t-test results in Table 1 demonstrate statistically significant differences (p-values < 0.05) in physical activity, type of food, HbA1c, hypertension, WBC, HDL, TC, TG, vitamin D, ferritin, systolic blood pressure, diastolic blood pressure, nationality, and BMI. This underscores their potential impact on the age at onset of T2D. In contrast, the variables occupation, smoking, gender, and marital status do not show statistically significant differences (p-value > 0.05).

**Fig 3:**
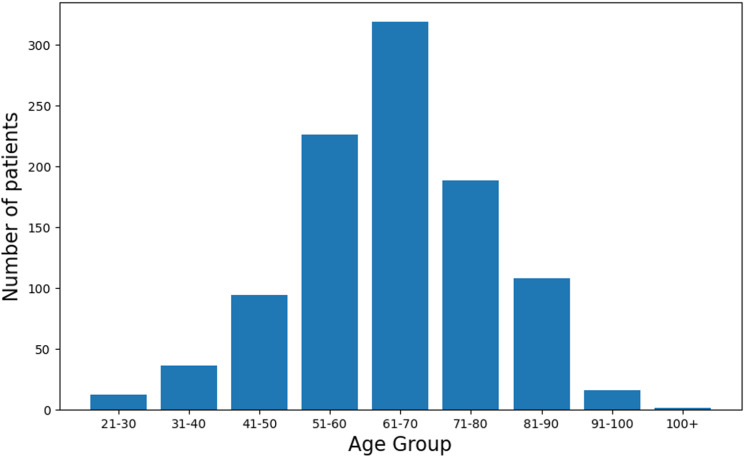
Distribution of age for all patients.

Table 2 presents the descriptive statistics for age within the studied cohort. The overall average age was 64.27 years, with a standard deviation of 13.31 years. The average age of males (n = 554) was 63.93 years, with a standard deviation of 12.03, while females (n = 446) had a slightly higher average age of 64.69 years and a greater standard deviation of 14.76 years. Both the median and mode for all the data were recorded at 65 years, indicating a central tendency towards this age. Based on Fig 3 the most common age group is 40 to 90 years old, as indicated by the highest number of patients in these age groups.

**Table 2:** Descriptive statistics for age.

	Both gender	Male	Female
**Number of Sample**	1000	554	446
**Average**	64.27	63.93	64.69
**St. Deviation**	13.31	12.03	14.76
**Median**	65	64	65
**Mode**	65	68	67
**Maximum**	104	104	99
**Minimum**	20	29	20

### 4.1. Multiple linear regression (MLR) development and comparison

To predict the age at onset of T2D in Saudi Arabia, MLR models were developed, with the age at onset serving as the dependent variable (y) and all variables listed in Table 1 as independent variables (x’s). We evaluated three different transformations of the dependent variable: the raw age at onset, the square root of the age, and the logarithmic transformation, in both models without interaction and models with interaction, to enhance the efficiency of the MLR models. Table 3 outlines the independent and interaction variables used in the MLR models: MLR4, MLR5, and MLR6. These models were selected using stepwise selection based on the smallest Akaike’s Information Criterion (AIC). MLR4 uses the age at onset as the dependent variable, MLR5 applies the square root transformation, and MLR6 uses the logarithmic transformation.

**Table 3:** Independent and interaction variables in selected MLR models.

Models of MLR	Independent and interaction variables
(a) MLR4: y	HbA1c + Gender + Smoking + Nationality + Hypertension + TF + BMI + SBP + WBC + DBP + HDL + Phy + TC + TG + Ferritin + VitaminD + Nationality:TG + Smoking:Nationality + HbA1c:Smoking + Gender:HDL + Hypertension:BMI + Gender:WBC + Hypertension:SBP + Smoking:WBC + Nationality:TC + TC:TG + TF:VitaminD + BMI:Ferritin + Phy: Smoking + WBC:VitaminD + TC:Ferritin + TF:HDL + SBP:HDL + HbA1c:TG + BMI:WBC + Nationality:TC:TG
(b) MLR5: sqrt(y)	HbA1c + Occupation + Gender + Smoking + Nationality + Hypertension + TF + BMI + SBP + WBC + DBP + Phy + HDL + TC + TG + Ferritin + VitaminD + TC:TG + TG:VitaminD + Hypertension:BMI + Nationality:TG + Gender:HDL + TC:Ferritin + HbA1c:Smoking + Smoking:Nationality + Hypertension:SBP + Smoking:SBP + Gender:WBC + Occupation:BMI + Occupation:SBP + BMI:DBP + Occupation:VitaminD + Occupation:TG + Hypertension:TG + WBC:VitaminD + TF:HDL + TF:VitaminD + Nationality:TC + Nationality:TC:TG
(c) MLR6:log(y)	HbA1c + MS + Occupation + Gender + Smoking + Nationality + Hypertension + TF + Phy + BMI + SBP + WBC + DBP + HDL + TC + TG + Ferritin + VitaminD + TG:Ferritin + TG:VitaminD + WBC:TC + Hypertension:BMI + BMI:HDL + Gender:HDL + Nationality:TG + TC:Ferritin + Smoking:WBC + Hypertension:TG + HbA1c:Smoking + Smoking:Nationality + BMI:DBP + Gender:WBC + Occupation:VitaminD + MS:DBP + TC:TG + TF:SBP + WBC:VitaminD + Occupation:TG + Occupation:BMI + MS:Nationality + Smoking:DBP + Nationality:WBC + Hypertension:SBP + Occupation:SBP + TF:HDL + TF:VitaminD + TC:TG:Ferritin + Smoking:Nationality:WBC

(:): interactions between variables, MS: Marital Status, Phy: Physical activity, TF: Type of food, HDL: High-Density Lipoprotein, TG: Triglycerides, TC: Total Cholesterol, WBC: White Blood Cells, BMI: Body Mass Index, SBP: Systolic Blood Pressure, DBP: Diastolic Blood Pressure.

Table 4 presents the performance metrics, including R^2^, RMSE, MAE, and accuracy, for the training and testing datasets. Among the models, MLR6 demonstrates the best performance, achieving an R^2^ of 0.91 for the training data and 0.90 for the testing data. Additionally, it recorded the lowest RMSE values of 0.14 (training) and 0.07 (testing) and the lowest MAE values of 0.11 (training) and 0.05 (testing), confirming its superior ability to predict the age at onset of T2D.

**Table 4:** MLR models for predicting age at onset of T2D.

Model	Training data			Testing data			
**Models without interaction**	$R^{2}$	**RMSE**	**MAE**	$R^{2}$	**RMSE**	**MAE**	**Accuracy**
MLR1	0.91	8.57	6.84	0.86	0.56	0.48	85%
MLR2	0.90	0.57	0.45	0.85	0.10	0.07	88%
MLR3	0.87	0.17	0.13	0.84	0.08	0.06	92%
**Models with interaction**	$R^{2}$	**RMSE**	**MAE**	$R^{2}$	**RMSE**	**MAE**	**Accuracy**
MLR4	0.91	8.2	6.57	0.89	0.55	0.45	88%
MLR5	0.91	0.53	0.42	0.90	0.14	0.08	94%
MLR6	0.91	0.14	0.11	0.90	0.07	0.05	96%

**MLR1:** Uses the original response variable y with the predictor variables (x′s).

**MLR2:** Uses the square root transformation (sqrt(y)) with the predictor variables (x′s).

**MLR3:** Uses the logarithmic transformation (logy) with the predictor variables (x′s).

**MLR4–MLR6:** Include interaction models in Table 3 with the predictor variables (x′s).

**Fig 4:**
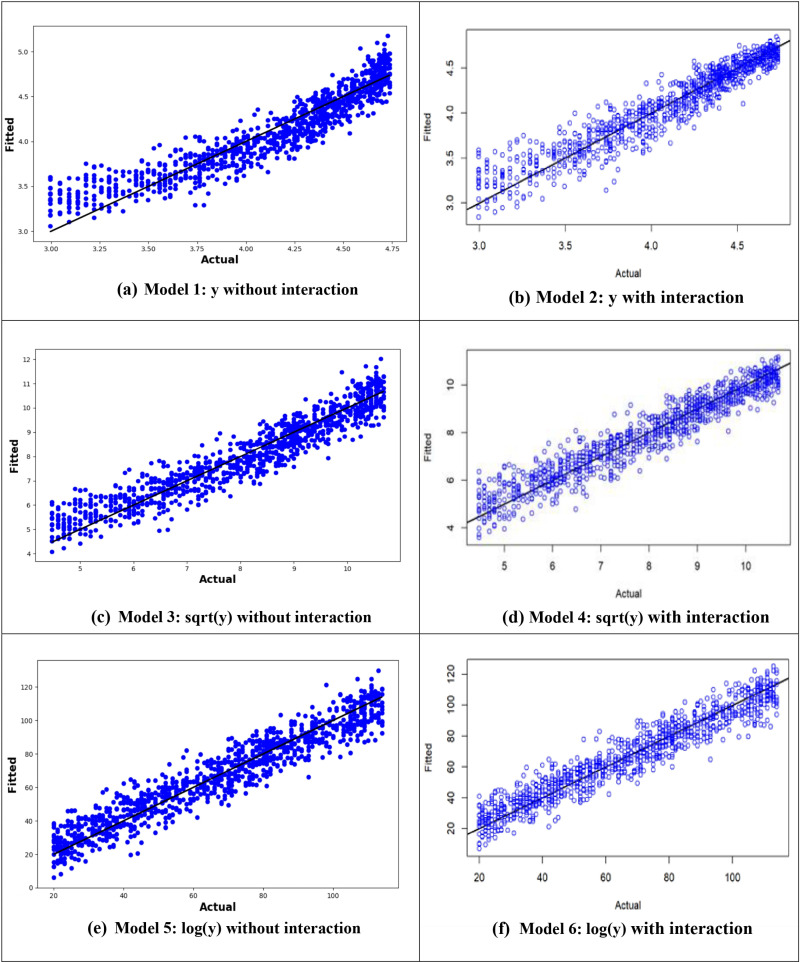
Plot of actual against predicted values for the MLR models for the original response variable (y), logarithmic transformation (logy), and square root transformation (sqrt(y)).

Fig 4 presents the actual versus predicted values plot, illustrating the predictive performance of the models. The plot shows that the MLR6 model, which utilizes the logarithm of the age at onset of T2D with interactions between variables, demonstrates the best predictive power due to the closeness of the predicted values to the actual values (Fig 4f).

### 4.2. Machine learning models ANN, RF, SVR, and DTR

This section delineates the modeling results for ANN, RF, SVR, and DTR, with the data partitioned into an 80/20 split between training and testing sets as depicted in Table 6. Each model was meticulously developed to predict the age at onset of T2D, denoted as (y), using the predictors specified in Table 1, excluding the age variable. To validate the robustness of these models, 10-fold cross-validation was implemented across all modeling approaches. Three different transformations of the target variable were systematically evaluated: the raw age at onset, its square root transformation, and its logarithmic transformation. The comparison of model performance was based on the test data set results. This rigorous testing was aimed to determine the most effective predictive approach.

**Fig 5:**
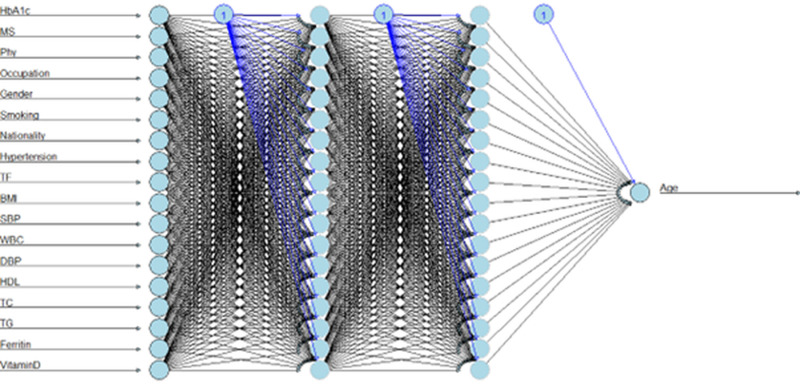
Neural network architecture. MS: Marital Status, Phy: Physical activity, TF: Type of food, HDL: High-Density Lipoprotein, TG: Triglycerides, TC: Total Cholesterol, WBC: White Blood Cells, BMI: Body Mass Index, SBP: Systolic Blood Pressure, DBP: Diastolic Blood Pressure.

In the ANN model, significant hyperparameters were finely tuned, including 18 input variables, two hidden layers with 18 neurons each, and a single output layer, through 10-fold cross-validation to optimize the network’s predictive accuracy, as shown in Fig 5. The figure illustrates the relationship between the number of hidden neurons and the ANN’s performance, where different numbers of neurons were tested in the hidden layers. Using 18 neurons in the hidden layers, the RMSE was significantly lower compared to values tested with fewer neurons. The RMSE for 10 neurons was about 10% higher than the RMSE for 18 neurons. The model performance in handling nonlinear relationships in the data improves through fine-tuning the hyperparameters. The logarithmic transformation (ANN3) proved to be the most effective, achieving *R*^2^ of 0.89, RMSE of 0.07, and MAE of 0.21, as presented in Table 5.

**Fig 6:**
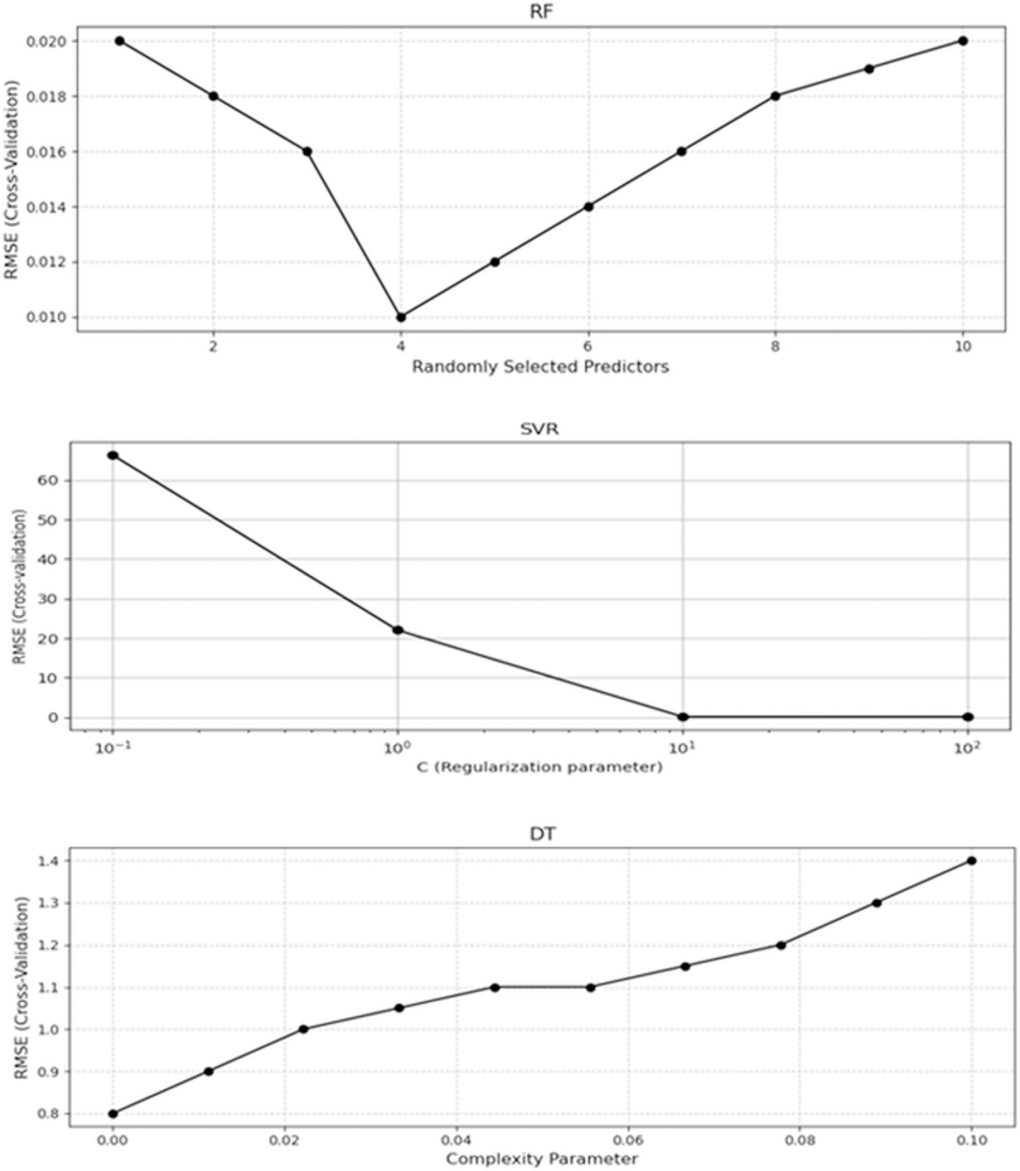
Optimal hyperparameters for RF, SVR, and DT models through cross-validation. The hyperparameter corresponding to the smallest RMSE presents the optimal value for each model.

The RF models were constructed using 500 decision trees to improve model performance. A crucial hyperparameter, which defines the number of variables randomly selected as candidates at each node, was critical in optimizing model performance. Through 10-fold cross-validation, it was revealed that the lowest RMSE was achieved when maximum features were set to 4 variables per node, as shown in Fig 6. In Fig 6, we can observe that as the number of variables selected per node increased beyond 4, the RMSE began to rise. When the maximum number of features was set to 6 or more, the RMSE increased from 0.010 to 0.014, indicating that the model’s performance worsened as more variables were considered at each node. This suggests that higher feature selection per node led to greater correlation between variables, which in turn caused the model to overfit and perform less accurately. On the other hand, setting the maximum number of features to 4 allowed the model to minimize correlation between variables, thus preventing overfitting and enhancing the model’s ability to generalize. This modification was crucial for achieving a balance between reducing variable correlation and improving model accuracy, thereby allowing the model to identify key patterns in the data without over-dependence on any individual feature. The ideal configuration of four features per node resulted in the highest model performance, yielding the lowest RMSE of 0.010, as illustrated in Fig 6. In Table 5, the logarithmic model (RF3) exhibited the best performance among the three transformations, with an R^2^ of 0.89, RMSE of 0.01, and MAE of 0.13.

**Table 5:** ANN, RF, SV, and DT models of age at onset of T2D.

Model	Training data			Testing data			
	$R^{2}$	RMSE	MAE	$R^{2}$	RMSE	MAE	Accuracy
ANN1: y	0.89	8.26	7.19	0.87	7.78	7.13	85%
ANN2: sqrt(y)	0.86	0.44	0.52	0.82	0.57	0.59	91%
ANN3: log(y)	0.86	0.05	0.17	0.89	0.07	0.21	95%
RF1: y	0.98	3.51	2.79	0.89	9.23	7.32	86%
RF2: sqrt(y)	0.98	0.23	0.18	0.88	0.61	0.48	93%
RF3: log(y)	0.98	0.06	0.04	0.89	0.01	0.13	96%
SVR1: y	0.90	7.42	6.76	0.81	7.96	7.01	85%
SVR2: sqrt(y)	0.89	0.32	0.36	0.84	0.36	0.47	93%
SVR3: log(y)	0.86	0.03	0.13	0.87	0.03	0.14	96%
DTR1: y	0.87	7.48	7.74	0.77	8.74	7.53	81%
DTR2: sqrt(y)	0.87	0.39	0.48	0.73	0.84	0.70	90%
DTR3: log(y)	0.86	0.03	0.13	0.75	0.05	0.18	95%

The hyperparameters in the SVR model with a linear kernel were finely tuned through 10-fold cross-validation. The analysis revealed that model performance significantly improved by modulating the cost parameter c. Fig 6 illustrates the effect of varying the value of c from 0.1 to 100 on the SVR’s performance. The figure demonstrates how RMSE decreases significantly as c increases, achieving the best performance when c ≈ 10^1^ (or c = 10). When c is small, the model tends to underfit, meaning it cannot capture the underlying patterns in the data, leading to high bias and RMSE. As c increases, the model fits the data better. However, when c becomes too large (e.g., 100), the model overfits, meaning it becomes too sensitive to noise in the training data, leading to high variance and worse performance on unseen data. The optimal value of c, around 10, balances bias, and variance, allowing the model to generalize well without overfitting or underfitting. The optimized model achieved an R^2^ of 0.87, RMSE of 0.03, and MAE of 0.14, outperforming other configurations, as detailed in Table 5.

The DTR model was developed by fine-tuning its hyperparameters, including the complexity parameter (cp), which controls the balance between model simplicity and accuracy. Using 10-fold cross-validation, it was observed that as the complexity parameter increased, the RMSE values steadily rose, as shown in Fig 6. The Fig. illustrates that with smaller cp values, the model tends to underfit the data, leading to a high RMSE. As cp increased, the RMSE decreased. However, as cp values became too large (e.g., 1 or greater), the RMSE values started rising again, reflecting that the model was overfitting the data. The optimal value for cp was observed to be 0.01, leading to the lowest RMSE of 0.03. In contrast, when cp was set to 0.1 or higher, the RMSE increased to 0.05 and 0.07, respectively, indicating that the model had become too complex and started to overfit the data. Among the applied transformations, the logarithmic model (DT3) achieved the best results, with *R*^2^ of 0.75, RMSE of 0.05, and MAE of 0.18, as presented in Table 5.

Across all models, the logarithmic transformation of the age at the onset of T2D consistently yielded superior results compared to the raw and square root transformations, making it the most effective transformation for predicting the onset age.

**Fig 7:**
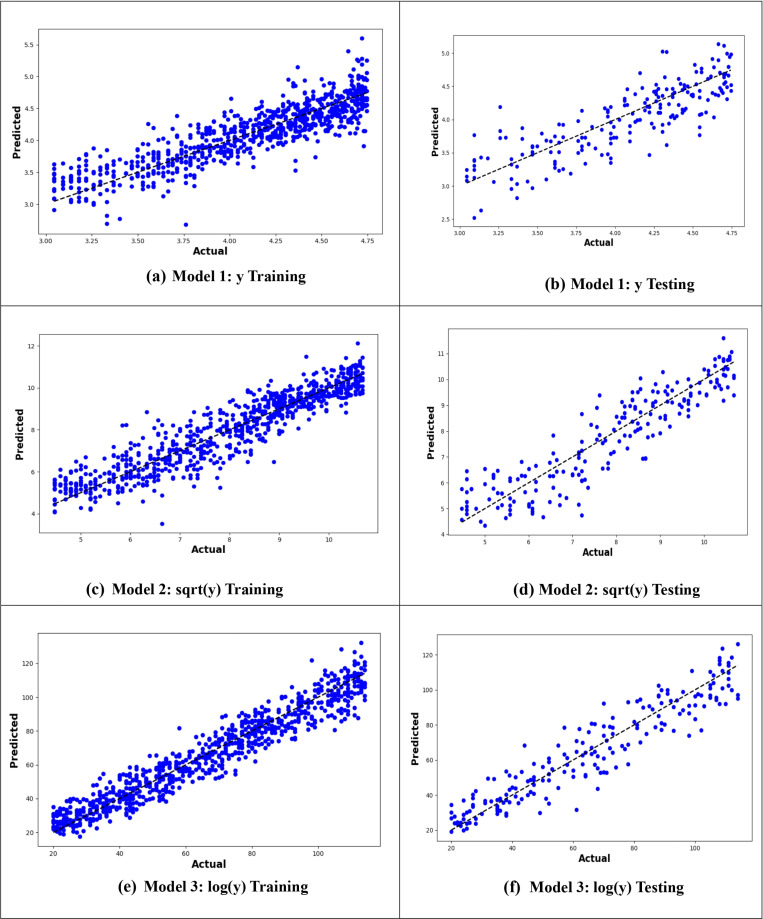
Plot of actual against predicted values for the ANN models for the original response variable (y), logarithmic transformation (logy), and square root transformation (sqrt(y)).

**Fig 8:**
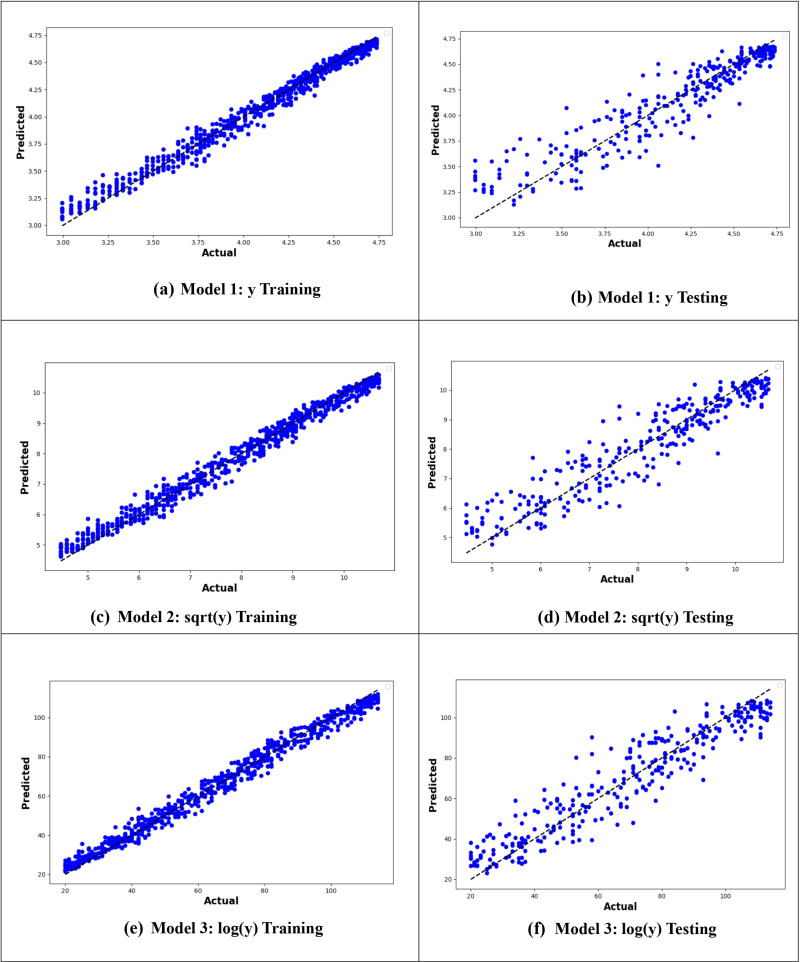
Plot of actual against predicted values for the RF models for the original response variable (y), logarithmic transformation (logy), and square root transformation (sqrt(y)).

**Fig 9:**
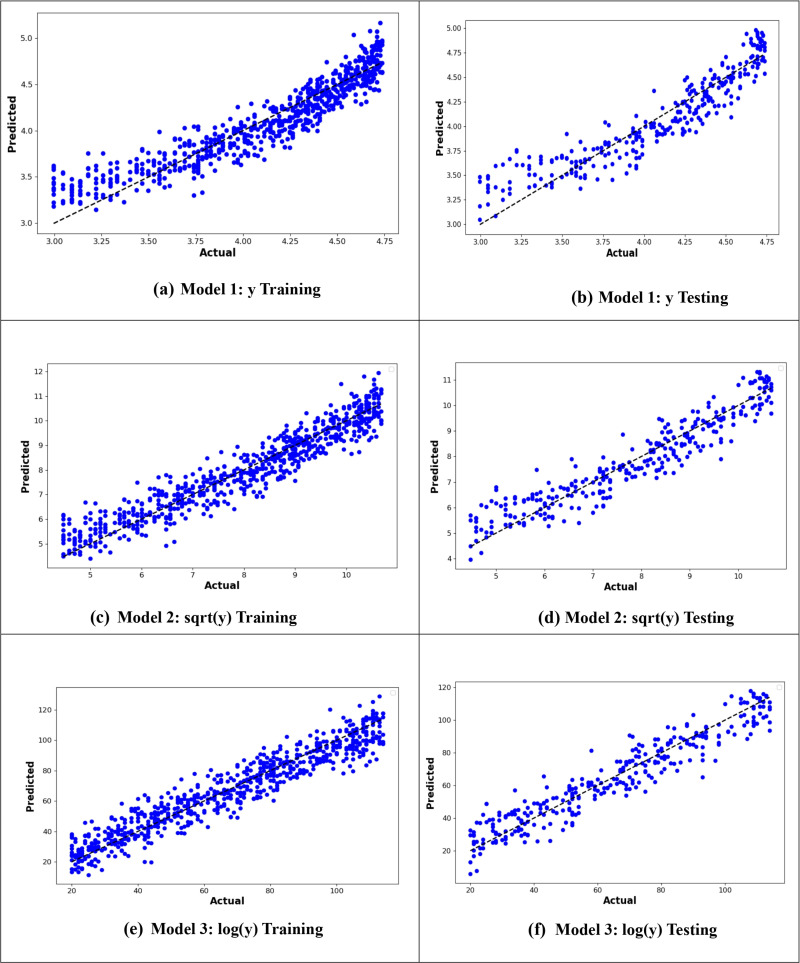
Plot of actual against predicted values for the SVR models for the original response variable (y), logarithmic transformation (logy), and square root transformation (sqrt(y)).

**Fig 10:**
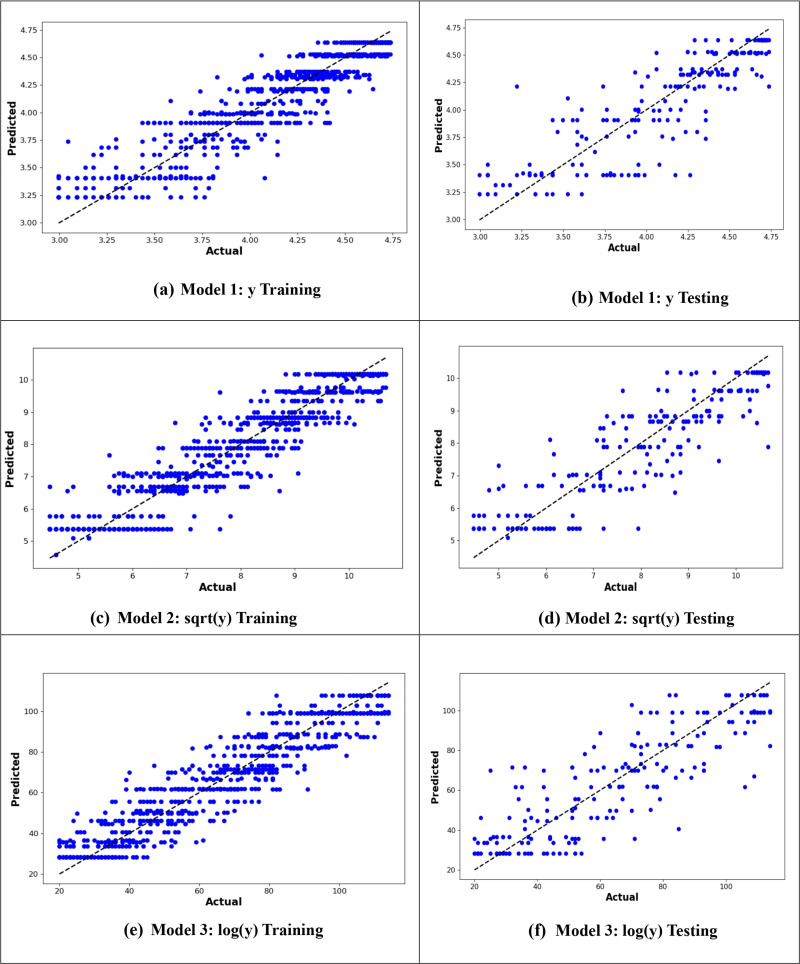
Plot of actual against predicted values for the DTR models for the original response variable (y), logarithmic transformation (logy), and square root transformation (sqrt(y)).

From –10, it is evident that the logarithmic transformation of the age at onset of T2D consistently provided the best alignment between observed and predicted values across all models. In Fig 7, the logarithmic ANN model (ANN3) demonstrated the closest fit, outperforming ANN1 and ANN2. Fig 8 highlights that the RF3 model, with the logarithmic transformation, achieved the best results, showing minimal error and a tight clustering of points around the line of perfect prediction. Similarly, Fig 9 indicates that SVR3 outperformed SVR1 and SVR2, offering a superior fit. Lastly, Fig 10 shows that DTR3 (logarithmic transformation) outperformed DTR1 and DTR2. Overall predictive performance of ANN, SVR, and DTR models was lower than that of the RF mode.

**Table 6:** The output of the MLR model.

Variable	Coefficient	95% C.I	p-value
Constant	1.7086	(0.1364, 1.5275)	2e-16 ^***^
HbA1c	−0.0070	(−0.0088, 0.4678)	0.1427
Marital Status	−0.0807	(−0.0903, 1.6600)	0.2141
Occupation	−0.0212	(0.0465, −1.7433)	0.0816
Gender	0.0028	(0.0372, 0.0785)	0.9379
Smoking	0.1006	(0.0810, 1.2433)	0.0079 ^**^
Nationality	−0.2097	(−0.0162, 0.3342)	0.0017^**^
Hypertension	0.0954	(0.0459, 0.0755)	0.3826
Type of Food	0.0520	(0.0159, 1.2666)	0.0011^**^
Physical activity	−0.2097	(−0.0467, 0.5685)	0.0214 ^*^
BMI	0.1172	(0.0150, 1.7875)	1.79e-14 ^***^
SBP	0.0561	(0.0179, 0.1386)	0.0017 ^**^
WBC	0.0963	(0.0223, 1.3162)	1.76e-05 ^***^
DBP	0.0473	(0.0190, 1.4872)	0.1305
HDL	0.0767	(0.0167, 1.5685)	5.57e-06 ^***^
TC	0.0936	(0.0260,0.5937)	0.0003 ^***^
TG	0.1310	(0.0301, 1.3532)	1.49e-05 ^***^
Ferritin	0.0549	(0.0255, 0.1556)	0.0314 ^*^
Vitamin D	0.0764	(0.0186, 1.0946)	4.60e-05 ^***^

*** Significant at p-value < 0.001.

** Significant at p-value < 0.01.

* Significant at p-value < 0.05.

Significant at p-value < 0.1.

### 4.3. Model validation

The model validation indicates that MLR and RF outperformed ANN, SVR, and DTR (Tables 4 and 5), with R^2^ values of 0.90 and 0.89, respectively, and the lowest RMSE and MAE (MLR: 0.07, 0.05; RF: 0.01, 0.13) when assessed using the test data set. Both models achieved 96% accuracy, confirming their superior ability to predict the age at onset of T2D in this study.

### 4.4. Risk factors associated with age at onset of T2D

The MLR model was employed to quantify the influence of each variable. Table 6 below summarizes the MLR model’s coefficients, confidence intervals (CI), and p-values, highlighting the significance of each factor in predicting the age at onset of T2D. This analysis shows that BMI, WBC, HDL, TG, vitamin D, and TC are key predictors of the age at onset of T2D, as they have highly significant p-values (p < 0.001). Additionally, factors such as nationality, smoking, type of food, physical activity, ferritin, and SBP also demonstrated statistically significant associations with T2D onset age (p < 0.05). In contrast, variables like HbA1c, marital status, occupation, gender, DBP and hypertension did not show statistically significant effects on T2D onset age in this model.

**Fig 11:**
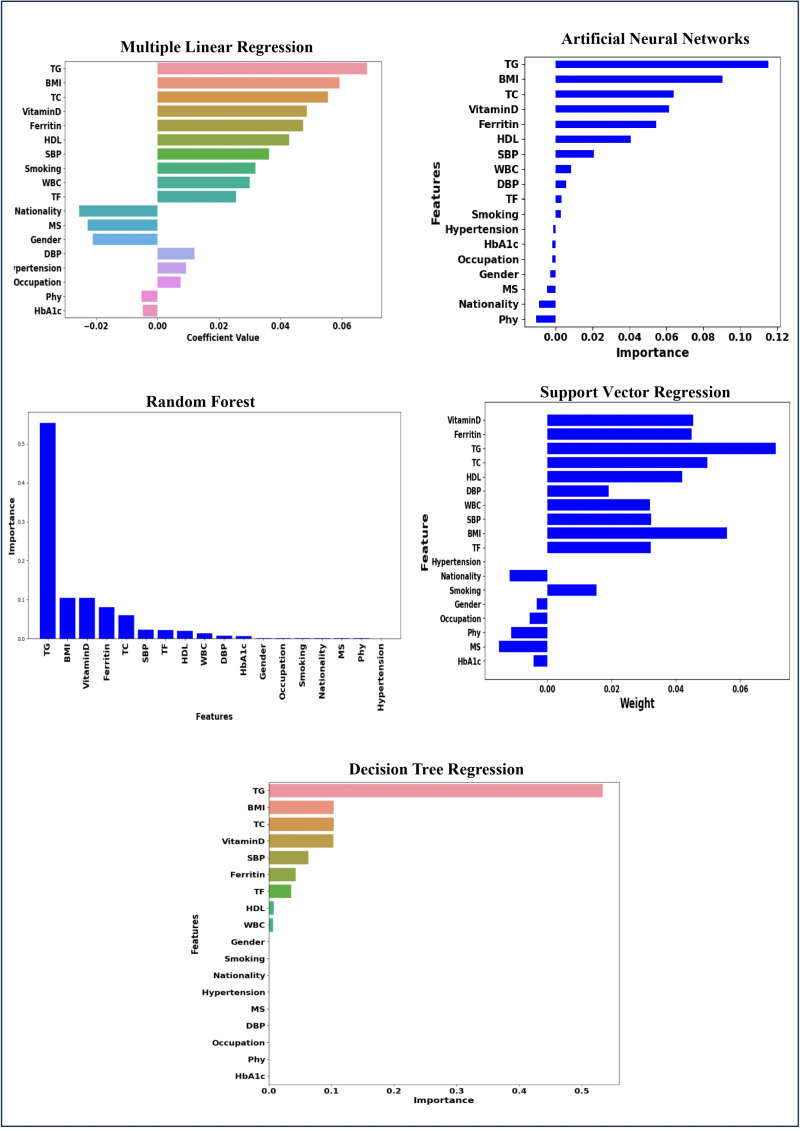
Risk factors of age at onset of T2D for MLR, ANN, RF, SVR, and DTR.

Fig 11 illustrates the variable importance across different models MLR, ANN, RF, SVR, and DTR. The key variables TG, TC, BMI, ferritin, HDL, SBP, WBC, and vitamin D emerge as the most influential factors across all models in predicting the age at onset of T2D. Despite slight variations in rankings, these variables are consistently identified as significant predictors in each model, indicating their strong association with T2D onset.

### 4.5. Modelling 40–90 years age group at onset of T2D

Studies from China, India, and the UK have shown higher T2D incidence in individuals over 40 [30,80,81]. This trend is also observed in our data. Fig 3 shows that T2D onset is most common between 40 and 90 years of age. Using MLR, ANN, RF, SVR, and DTR models, we analyzed key demographic, lifestyle, and lipid profile variables in 766 patients in this age group (average age: 63 years). The sample included 173 males, 593 females, 647 obese individuals, 576 smokers, and 594 who were inactive. Lipid analysis revealed an average HDL of 2.97 (634 with low HDL), TG of 4.35 (560 elevated), and TC of 4.63 (569 elevated), indicating widespread lipid abnormalities.

MLR models were developed to predict the age at onset of T2D for this age group, incorporating interactions between the variables listed in Table 7. Table 8 compares models without interactions (MLR7-MLR9) with those with interactions (MLR10-MLR12), based on R^2^, RMSE, and MAE metrics. For the without interactive models (MLR7-MLR9), the R^2^ was (0.85) with RMSE values of (0.51, 0.12, and 0.08) and MAE values of (0.43, 0.23, and 0.12) respectively. Conversely, for the interactive models (MLR10-MLR12), the R^2^ was (0.87) with RMSE values of (0.46, 0.07, and 0.06) and MAE values of (0.44, 0.18, and 0.11) respectively.

**Table 7:** Variables in selected MLR models in age group (40–90).

Models of MLR	Independent and interaction variables
(a) MLR10: y	HbA1c + MS + Gender + Smoking + Nationality + TF + BMI + SBP + WBC + DBP + HDL + TC + TG + Phy + Ferritin + VitaminD + HbA1c: Smoking + Gender: HDL + Smoking:Nationality + Gender:WBC + Nationality:TG + MS:VitaminD + Nationality:TF + HbA1c:HDL + MS:BMI + MS:Ferritin + BMI:Ferritin + TC:Ferritin + TF:VitaminD + TF:SBP + TF:HDL + HDL:Ferritin + DBP:TG + Nationality:TC
(b) MLR11: sqrt(y)	HbA1c + MS + Occupation + Gender + Smoking + Nationality + TF + BMI + SBP + WBC + DBP + HDL + Phy + TC + TG + Ferritin + VitaminD + TC:TG + HbA1c:Smoking + Smoking:Nationality + Gender:HDL + TF:HDL + Gender:WBC + MS:BMI + Nationality:TG + Nationality:TC + Occupation:TG + Occupation:BMI + Phy:SBP + Occupation:Nationality + Nationality:DBP + BMI:Ferritin + TC:Ferritin + TF:VitaminD + HbA1c:HDL + Nationality:TF + MS:Occupation + MS:VitaminD + MS:Ferritin + Nationality:TC:TG
(c) MLR12: log(y)	HbA1c + MS + Occupation + Gender + Smoking + Nationality + TF + BMI + SBP + WBC + DBP + HDL + Phy + TC + TG + Ferritin + VitaminD + TC:TG + TF:SBP + HbA1c:Smoking + TC:Ferritin + Smoking:Nationality + MS:BMI + Gender:WBC + TF:HDL + Gender:HDL + Occupation:TG + Occupation:BMI + Nationality:TG + Nationality:TC + Occupation:Nationality + Nationality:DBP + Occupation:SBP + HbA1c:Occupation + Smoking:SBP + DBP:TG + MS:Occupation + Nationality:TC:TG

(:): interactions between variables, MS: Marital Status, Phy: Physical activity, TF: Type of food, HDL: High-Density Lipoprotein, TG: Triglycerides, TC: Total Cholesterol, WBC: White Blood Cells, BMI: Body Mass Index, SBP: Systolic Blood Pressure, DBP: Diastolic Blood Pressure.

**Table 8:** MLR models of age at onset of T2D of age group (40–90).

Model	Training data			Testing data			
**Models without interaction**	$R^{2}$	**RMSE**	**MAE**	$R^{2}$	**RMSE**	**MAE**	**Accuracy**
MLR7	0.85	8.37	6.76	0.85	0.51	0.43	83%
MLR8	0.85	0.48	0.39	0.85	0.12	0.23	85%
MLR9	0.85	0.09	0.24	0.85	0.08	0.12	88%
**Models with interaction**	$R^{2}$	**RMSE**	**MAE**	$R^{2}$	**RMSE**	**MAE**	**Accuracy**
MLR10	0.87	7.98	6.40	0.87	0.46	0.44	84%
MLR11	0.87	0.45	0.36	0.87	0.07	0.18	92%
MLR12	0.87	0.07	0.22	0.87	0.06	0.11	97%

**MLR7:** Uses the original response variable y with the predictor variables (x′s).

**MLR8:** Uses the square root transformation (sqrt(y)) the predictor variables (x′s).

**MLR9:** Uses the logarithmic transformation (logy) with the predictor variables (x′s).

**MLR10–MLR12:** Include interaction models in Table 7 with the predictor variables (x′s).

Among these, the best model for predicting the age at onset of T2D in this group was MLR12, the logarithmic transformation model with interactions between variables. This model demonstrated the highest performance, evidenced by a high R^2^ value and the lowest RMSE and MAE values, as shown in Table 8.

**Fig 12:**
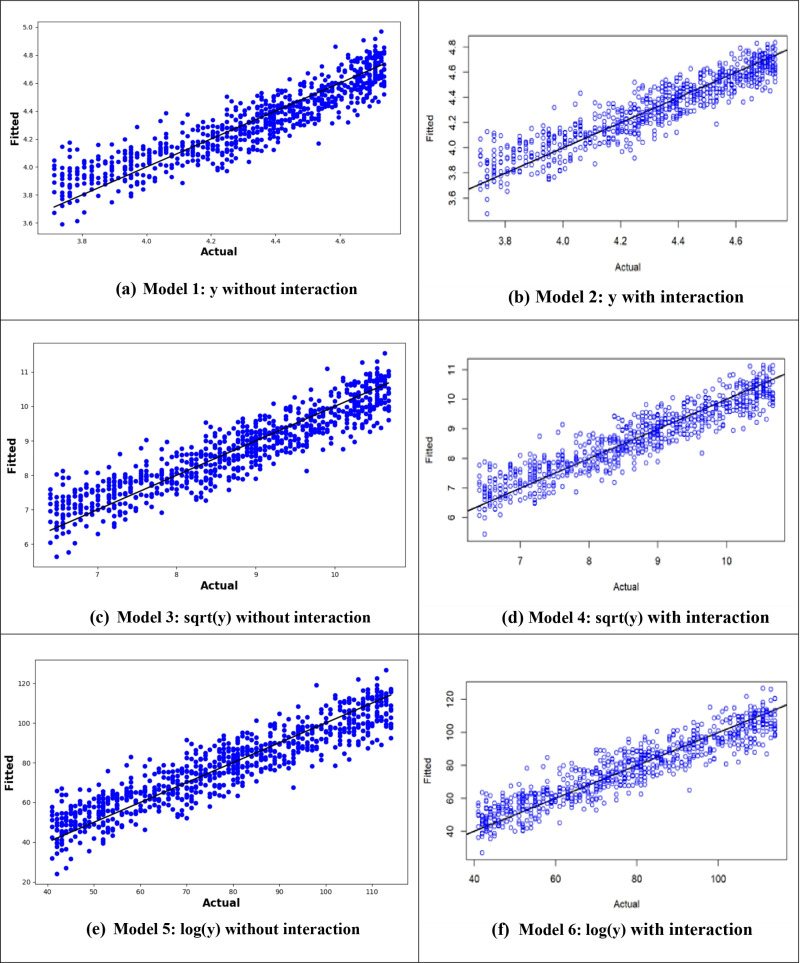
Plots of the MLR models’ training and testing data in the 40–90 years age group. (a, b) *y*: age at onset(c, d) y: square root of age at onset(e, f) y: logarithm of age at onset.

Fig 12 illustrates the comparison between observed and fitted values for the MLR models, both with and without interactions, for individuals aged 40–90. The graph shows that the MLR12 model, which applies the logarithm of the age at onset of T2D along with variable interactions is the best in terms of predictive performance due to the minimal residuals between the predicted and actual values (Fig 12f). However, despite the improved performance (with interactions), the age-specific MLR model achieved similar robustness as the model applied to the entire dataset, with a minor difference in R^2^ values (0.87 vs. 0.90).

Table 9 compares the performance of several models, including ANN, RF, SVR, and DTR, in predicting the age at onset of T2D for individuals aged 40–90. The RF models, particularly RF6 (logarithmic transformation), outperformed the others, achieving the highest R^2^ (0.97 for training, 0.87 for testing) and the lowest RMSE and MAE (0.12 and 0.04, respectively).

**Table 9:** ANN, RF, SV, and DT models of age at onset of T2D for age group (40–90).

Model	Training data			Testing data			
	$R^{2}$	RMSE	MAE	$R^{2}$	RMSE	MAE	Accuracy
ANN4: y	0.89	4.81	5.45	0.63	7.68	8.85	88%
ANN5: sqrt(y)	0.90	0.16	0.31	0.65	0.34	0.25	92%
ANN6: log(y)	0.94	0.03	0.14	0.67	0.08	0.22	94%
RF4: y	0.97	3.62	2.89	0.81	9.02	7.19	89%
RF5: sqrt(y)	0.97	0.2	0.16	0.83	0.53	0.42	94%
RF6: log(y)	0.97	0.12	0.1	0.87	0.04	0.13	97%
SVR4: y	0.84	7.04	6.63	0.83	7.82	7.07	89%
SVR5: sqrt(y)	0.84	0.23	0.39	0.83	0.26	0.41	94%
SVR6: log(y)	0.84	0.01	0.09	0.83	0.07	0.23	97%
DTR4: y	0.85	6.9	6.5	0.68	4.85	9.54	79%
DTR5: sqrt(y)	0.84	0.23	0.38	0.66	0.55	0.58	90%
DTR6: log(y)	0.83	0.01	0.09	0.7	0.05	0.17	92%

**Fig 13:**
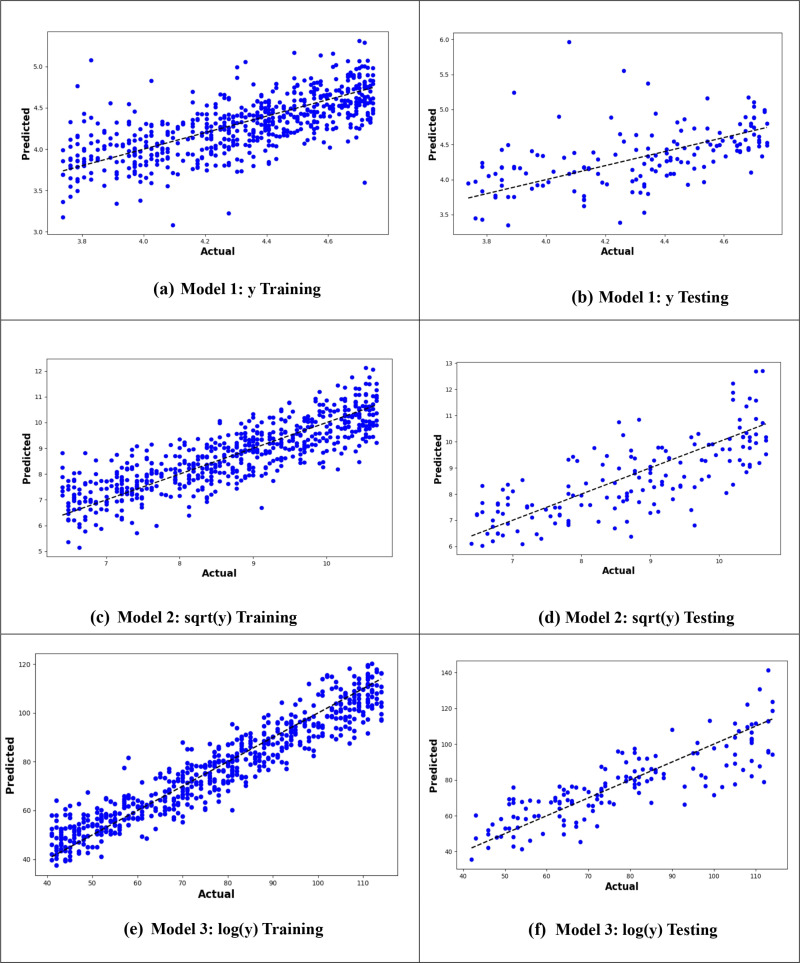
Plots of the ANN models’ training and testing data in the 40–90 years age group. (a, b) *y*: age at onset(c, d) y: square root of age at onset(e, f) y: logarithm of age at onset.

**Fig 14:**
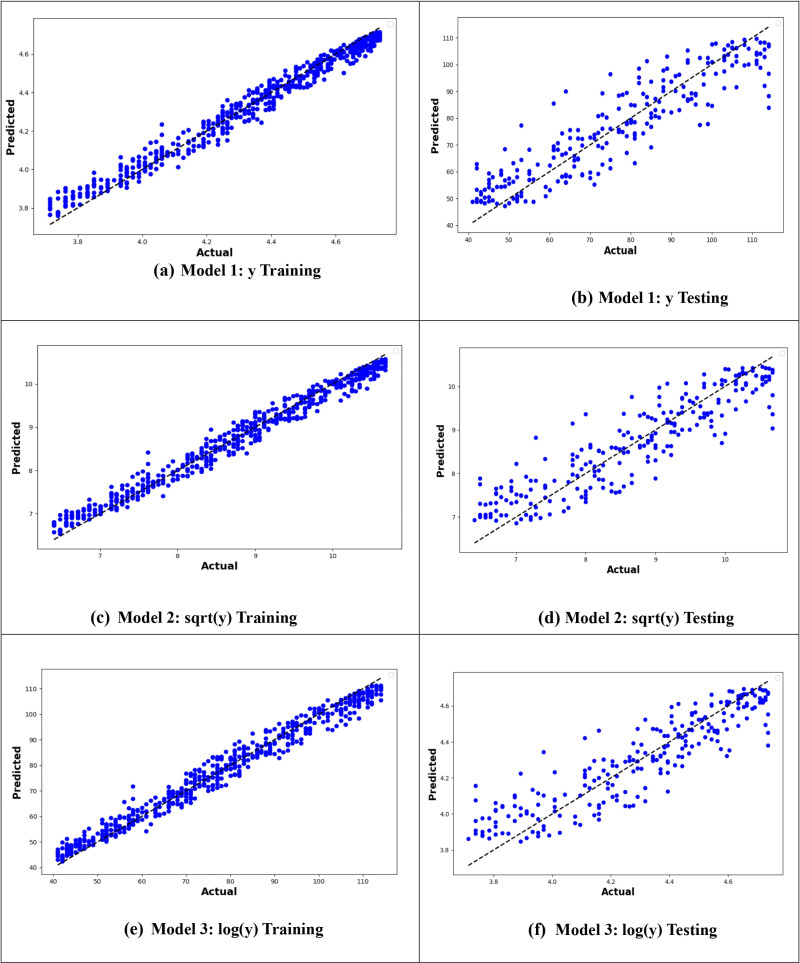
Plots of the RF models’ training and testing data in the 40–90 years age group. (a, b) *y*: age at onset(c, d) y: square root of age at onset(e, f) y: logarithm of age at onset.

**Fig 15:**
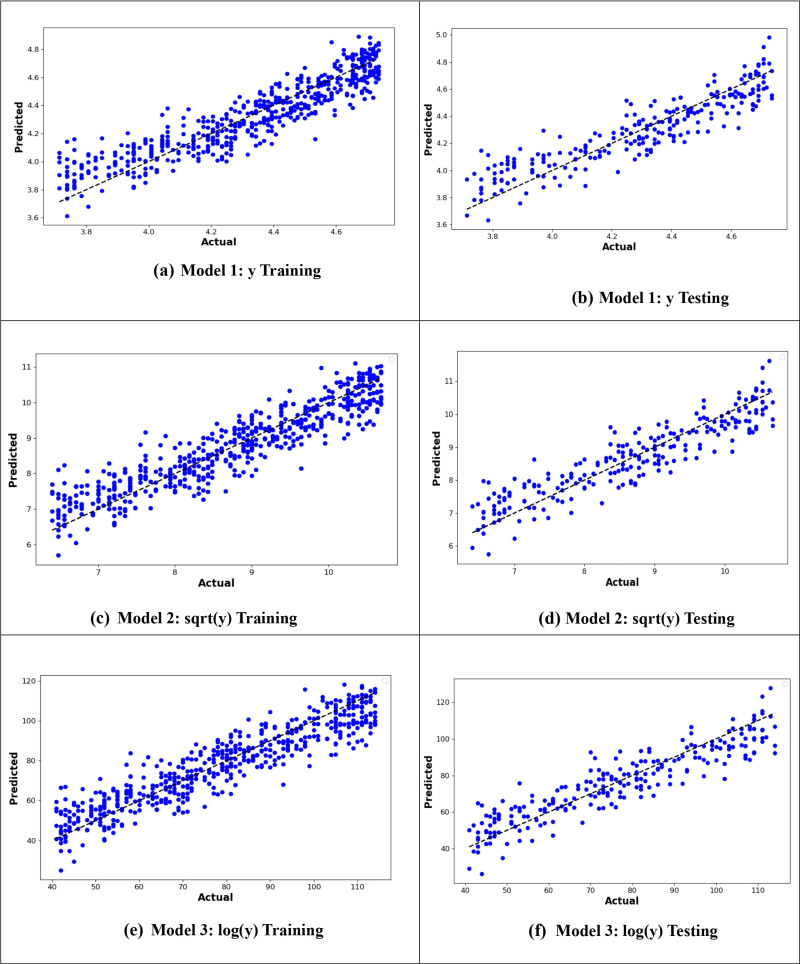
Plots of the SVR models’ training and testing data in the 40–90 years age group. (a, b) *y*: age at onset(c, d) y: square root of age at onset(e, f) y: logarithm of age at onset.

**Fig 16:**
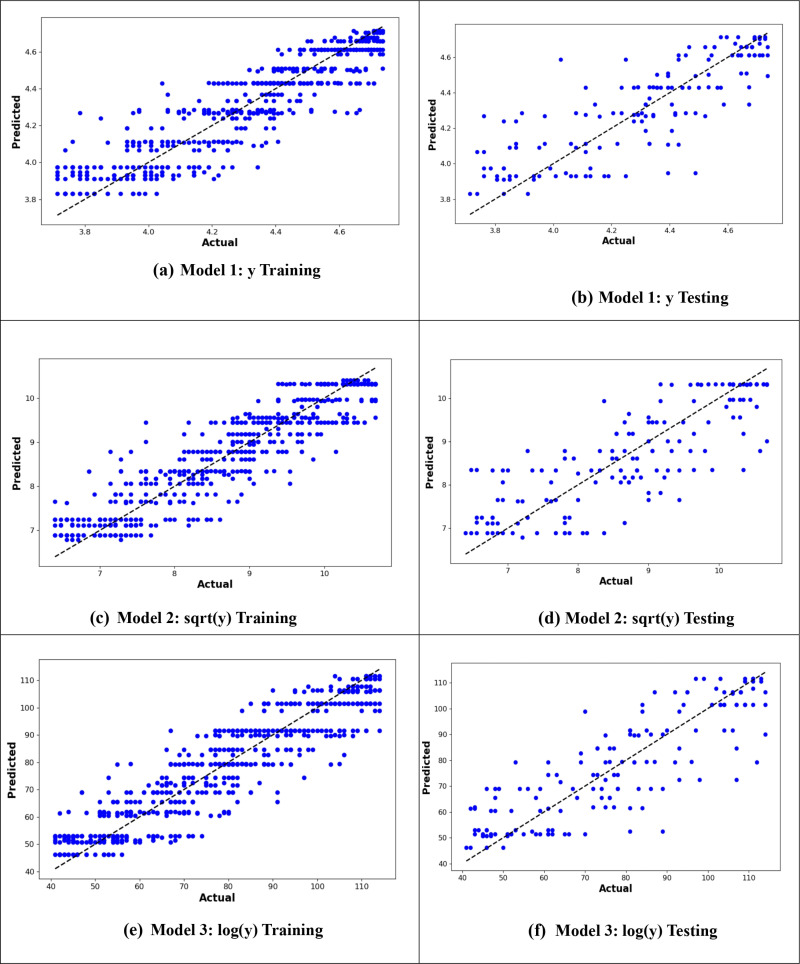
Plots of the DTR models’ training and testing data in the 40–90 years age group. (a, b) *y*: age at onset(c, d) y: square root of age at onset(e, f) y: logarithm of age at onset.

Figs 13–16 compare the actual values against the expected values for ANN, RF, SVR, and DTR models in predicting the age at onset of T2D for the 40–90 age group. The logarithmic transformations consistently performed better across all models. However, the RF model in Fig 13 demonstrated the highest predictive accuracy, with minimal error and the tightest clustering of points around the line of perfect prediction, showing the closest alignment between observed and predicted values.

#### 4.5.1. Model validation.

The model validation (Tables 8 and 9), using a 10-fold cross-validation dataset split 80/20 for training and testing with a fixed random state for reproducibility, shows that using the test data set, MLR and RF outperformed ANN, SVR, and DTR, both achieving an R^2^ of 0.87. MLR recorded an RMSE of 0.06 and an MAE of 0.11 but RF achieved the best performance with an RMSE of 0.04, an MAE of 0.13, and 97% accuracy, confirming its superior predictive ability for T2D age onset.

#### 4.5.2. Significant predictors for the 40–90 years age group.

**Fig 17:**
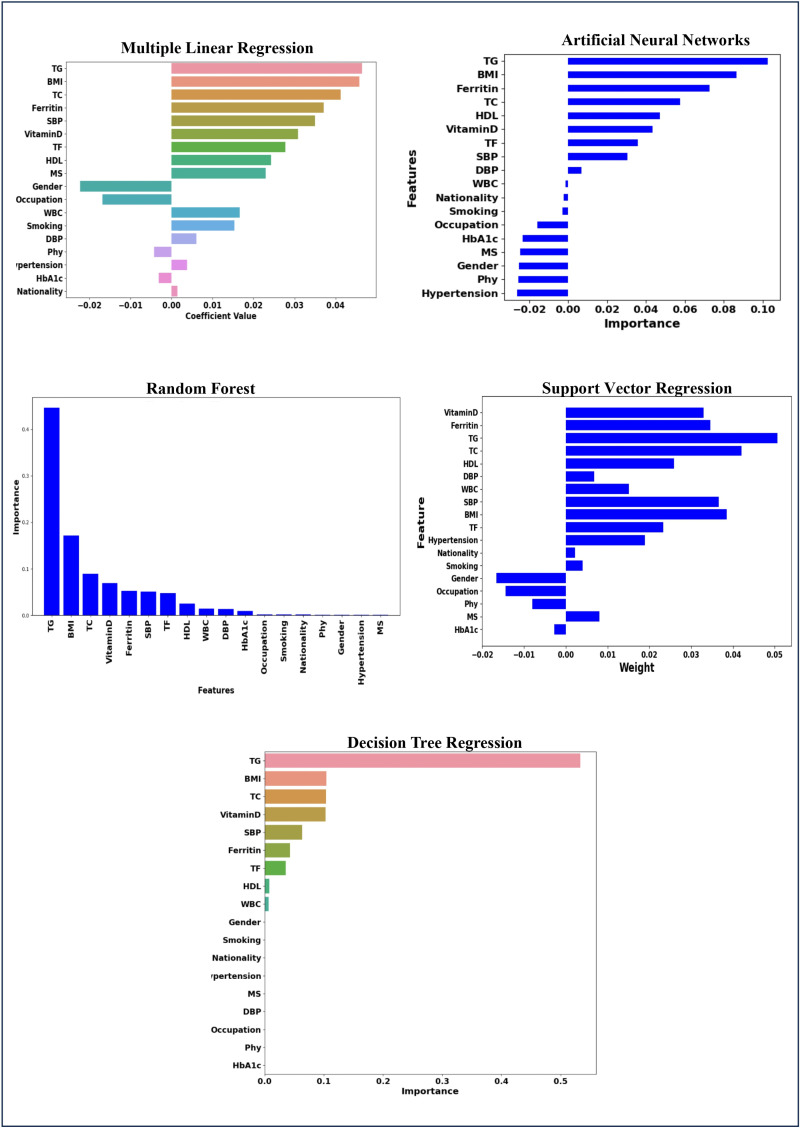
Risk factors of T2D for age group (40–90) identified by all MLR, ANN, RF, SVR, and DTR.

Table 10 presents the output of the MLR model for the 40–90 age group, highlighting the strongest predictors of T2D onset age, including type of food, BMI, SBP, WBC, TG, ferritin, and vitamin D, all with p-values less than 0.001. Smoking, gender, hypertension, DBP, TC, and HDL also are significant predictors (p-value < 0.05). The nutrient intake, lipid profile, blood pressure, and inflammation markers like WBC and ferritin were the most influential factors in predicting the onset of T2D in this age group. The performance of these variables in the other models is presented in Fig 17.

**Table 10:** The output of the MLR model of (40–90) age group.

Variable	Coefficient	95% C.I	p-value
Constant	2.6624	(0.5179, 2.5276)	2e-16 ^***^
HbA1c	−0.0656	(−0.0768, 1.4679)	0.6028
Marital Status	0.4127	(0.2182, 2.6607)	0.0688
Occupation	−0.3202	(−0.5130, 1.7435)	0.7092
Gender	−0.0509	(−0.0672, 0.1567)	0.03060 ^*^
Smoking	0.58199	(0.2085, 1.2437)	0.0379 ^*^
Nationality	0.7016	(0.4126, 1.3345)	0.1603
Hypertension	0.0754	(0.0859, 2.0757)	0.0382 ^*^
Type of Food	0.1685	(0.0734, 1.2663)	20e-09 ^***^
BMI	0.0319	(0.0660, 1.7877)	2.63e-14 ^***^
SBP	0.2565	(0.0556, 0.2887)	3.64e-06 ^***^
WBC	0.1471	(0.0339, 1.3166)	5.52e-06 ^***^
DBP	0.0003	(0.0280, 0.4877)	0.0233 ^*^
HDL	0.2599	(0.0875, 0.5686)	0.0052 ^**^
TC	0.2221	(0.0813,1.5933)	0.0024 ^**^
TG	0.3004	(0.0861, 1.3534)	3.91e-05 ^***^
Ferritin	0.2022	(0.0747, 0.1553)	1.07e-05 ^***^
Vitamin D	0.02704	(0.0177, 0.0946)	4.61e-16 ^***^

*** Significant at p-value < 0.001.

** Significant at p-value < 0.01.

* Significant at p-value < 0.05.

Significant at p-value < 0.1.

## 5. Discussion

In this study, de-identified data from 1000 patients diagnosed with T2D at King Abdulaziz University Hospital were used to predict the age at onset of T2D. The data indicated an increased prevalence among males in all age groups, while the highest prevalence among females was noted in the most prevalent age category (40–90 years). This pattern aligns with trends reported in East Asian studies [82], but contrasts with findings from America [27], where the highest incidence rates were noted in the 30–39 age group. This variation may be attributed to differences in lifestyle patterns, cultural norms, and environmental factors. In this study, a variety of advanced analytical models including Multiple Linear Regression (MLR), Artificial Neural Networks (ANN), Random Forest (RF), Support Vector Regression (SVR), and Differential Tree Regression (DTR) were employed to predict the age at onset of T2D and to examine the impact of certain variables on T2D.

Statistical analysis demonstrated that the MLR and RF models significantly outperformed the ANN, SVR, and DTR models in predicting the onset age of T2D. Respectively, the RF and MLR models achieved R^2^ (0.89, 0.90), RMSE (0.01, 0.07), and MAE (0.13, 0.05), with a prediction accuracy of 96% across all data. For the age group of (40–90) years, both models exhibited outstanding performance, with RF and MLR recording R^2^ (0.87, 0.87), RMSE (0.04, 0.06), and MAE (0.13, 0.11). This performance aligns with findings from previous studies, such as the research conducted in Slovenia, which demonstrated the superiority of the Random Forest model in predicting T2D onset [33]. Other studies also showed the efficiency of both Random Forest (RF) and Multiple Linear Regression (MLR) in predicting the age at onset of different diseases [36,37] such as childhood T1D and childhood obesity. This further emphasizes the reliability and versatility of these models in predicting age at the onset of diseases. These results underscore the high dependability of the RF and MLR models, highlighting their significant potential in predicting the age at onset, especially when dealing with health data.

The models identified key risk factors that influence the onset age of T2D, including triglycerides (TG), total cholesterol (TC), high-density lipoprotein (HDL), ferritin, body mass index (BMI), systolic blood pressure (SBP), white blood cell (WBC) count, diet type, and vitamin D levels. The findings revealed extremely high levels of TG and TC, accompanied by a significant reduction in HDL levels, which are critical markers associated with the early onset of T2D and an increased risk of cardiovascular diseases. These findings are consistent with previous studies [28,30], which demonstrated a strong correlation between elevated TG and TC levels, decreased HDL levels, and an increased risk of T2D. These results underscore the importance of targeted interventions to address these risk factors to mitigate the progression of T2D and its associated complications. Additionally, low levels of ferritin and vitamin D were prevalent in the sample, suggesting that nutritional deficiencies and inflammatory markers could contribute to the early onset of T2D. These factors significantly increase the risk of developing T2D at an earlier age. These findings align with those reported in the literature [26,27,29], which identified the significant impact of BMI and WBC on the risk of developing T2D. The strong correlations observed between these health markers and T2D at its early stages emphasize the critical need for targeted lifestyle and nutritional interventions among high-risk populations.

Furthermore, accurately estimating the likely age of onset enables earlier treatment initiation, which can significantly reduce the complications associated with delayed diagnoses. This underscores the importance of applying the findings of this study to health practices in Saudi Arabia to improve prevention and early intervention strategies.

## 6. Conclusions

Type 2 diabetes poses a major global public health threat. In Saudi Arabia, the prevalence of diabetes presents a significant challenge, as the country ranks seventh globally in terms of diabetes incidence. The present study aims to fill the gap in the literature by proposing an optimal predictive model for the age at onset of T2D among citizens within the kingdom using a rich and diverse dataset. Based on the available information, this study is among the first to utilize multiple machine learning models, including MLR, ANN, RF, DT, and SVR, to predict the age at onset for T2D in Saudi Arabia and to identify the most significant factors contributing to its development.

The findings revealed that the RF model demonstrated the best performance, achieving the highest R^2^ and the lowest values for RMSE and MAE, particularly for individuals aged 40 to 90. Additionally, the MLR model showed strong performance when a logarithmic transformation of the age at onset was applied. Key predictors identified included lipid profiles such as triglycerides (TG), total cholesterol (TC), high-density lipoprotein (HDL), body mass index (BMI), systolic blood pressure, and white blood cell count, along with lifestyle factors related to smoking and dietary habits.

These results contribute to our understanding of T2D in Saudi Arabia and aid in developing strategies to identify individuals at higher risk for early intervention. Moreover, the study underscores the importance of increasing awareness about healthy lifestyle habits and nutrition, as well as the need for public health initiatives and policies to curb the spread of the disease. To further address the limitations of this study, other risk variables, such as genetic factors, complications, medications, and inflammatory factors, should be explored to provide a more comprehensive understanding of the factors influencing the age at onset of T2D. Additionally, including more cities and regions would enhance the diversity and size of the dataset, contributing to the development of a more robust predictive framework.
